# A Survey of Residents' Perceptions of the Effect of Large-Scale Economic Developments on Perceived Safety, Violence, and Economic Benefits

**DOI:** 10.1155/2015/903264

**Published:** 2015-07-27

**Authors:** Anthony Fabio, Ruth Geller, Michael Bazaco, Todd M. Bear, Abigail L. Foulds, Jessica Duell, Ravi Sharma

**Affiliations:** ^1^Epidemiology Data Center, Department of Epidemiology, Graduate School of Public Health, University of Pittsburgh, Pittsburgh, PA 15261, USA; ^2^Department of Behavioral and Community Health Sciences, Graduate School of Public Health, University of Pittsburgh, Pittsburgh, PA 15261, USA

## Abstract

*Background*. Emerging research highlights the promise of community- and policy-level strategies in preventing youth violence. Large-scale economic developments, such as sports and entertainment arenas and casinos, may improve the living conditions, economics, public health, and overall wellbeing of area residents and may influence rates of violence within communities. *Objective*. To assess the effect of community economic development efforts on neighborhood residents' perceptions on violence, safety, and economic benefits. *Methods*. Telephone survey in 2011 using a listed sample of randomly selected numbers in six Pittsburgh neighborhoods. Descriptive analyses examined measures of perceived violence and safety and economic benefit. Responses were compared across neighborhoods using chi-square tests for multiple comparisons. Survey results were compared to census and police data. *Results*. Residents in neighborhoods with the large-scale economic developments reported more casino-specific and arena-specific economic benefits. However, 42% of participants in the neighborhood with the entertainment arena felt there was an increase in crime, and 29% of respondents from the neighborhood with the casino felt there was an increase. In contrast, crime decreased in both neighborhoods. *Conclusions*. Large-scale economic developments have a direct influence on the perception of violence, despite actual violence rates.

## 1. Introduction

Over the past 15 years, U.S. has witnessed a general decline in overall rates of youth homicide [1]. Nonetheless, youth violence rates remain high in this country, with homicide being the third leading cause of death among persons aged 10–24 years [2]. Youth violence can affect communities by substantially increasing the cost of health care, reducing productivity, and diminishing property values [3]. In 2000, it was estimated that the medical care and lost productivity costs associated with youth violence were more than $70 billion [4].

Youth violence has been linked to a variety of factors including individual, family, community, and societal characteristics. Community-level risk can have negative influences even on youth who are not exposed to individual- or family-level risk factors. Major community risk factors for violence include high density of alcohol outlets, community norms favorable toward violence, residential instability, transitions and mobility, low neighborhood attachment, social disorganization, the presence of gangs, and extreme economic deprivation [5, 6].

Although much research has been conducted on interventions to change the characteristics of individuals and families, less has focused on evaluating interventions and policies designed to change community economic conditions or characteristics of the physical environment. Studies have been conducted on the impact of interventions on individual-level variables; however, evaluations at the community level have been sparse. Some research is emerging that highlights the promise of community- and policy-level strategies in preventing youth violence. For example, modifications to the built environment—such as improvements to the pedestrian environment and architectural changes—can contribute to relative reductions in 911 calls and crimes, possibly through increases in social capital and intolerance of criminal activity [7, 8]. It is theorized that large-scale economic developments, such as sports and entertainment arenas and casinos, improve the living conditions, economics, public health, and overall wellbeing of area residents and may influence rates of violence within communities [9, 10]. Local government and business developers often suggest that building an arena or a casino in a neighborhood may provide potential societal benefits as a result of the construction and the business drawn in; thus, many new arenas are placed in areas of needed economic growth. However, scientific research to support these ideas is scarce, and there is much debate about the true benefits of economic developments [10]. Research consistently suggests that economically disadvantaged neighborhoods have poorer public health outcomes, including higher rates of violent crime, chronic disease, and risky behavior, and that there is large heterogeneity with regard to these factors across neighborhoods within a city [11, 12]. According to Siegfried and Zimbalist, “independent work on the economic impact of stadiums and arenas has uniformly found that there is no statistically significant positive correlation between sports facility construction and economic development” [13]. This has been substantiated by cross-sectional and time series analyses [13]. Very little work has assessed the effect of sports facility construction on violence outcomes. The state of research is similar regarding the benefits of casinos [10]. Whether casino development causes economic growth in surrounding neighborhoods is a complex question, and the literature is not consistent in its conclusions [9, 10]. The few studies that have assessed economic growth looked at large geographic areas such as the county or state. For example, [14] describes that after the licensing of casinos in Monaco, Nevada, and Atlantic City, these cities grew dramatically economically and became destinations for tourism. Also, few studies have looked at the effects of casinos on crime and delinquency, but, of those that have, the findings have been inconsistent [15, 16]. There is a need for a more focused, community-level survey of resident perceptions regarding the effect of these major community development projects on perceived safety, violence, and economic benefits.

A consistent literature suggests that the perception of crime is often different from actual crime. For instance, in a study conducted in Australia, results suggested that people often exaggerated the risks of becoming a victim of crime [17]. This was corroborated in two subsequent studies [18]. In another study conducted in New Zealand, crime in an individual's own neighborhood influenced fear of crime, but crime occurring in neighboring communities had little effect on perceived safety [19]. This difference in perception is referred to as the “paradox of fear” [20, 21]. Age, gender, and race have been demonstrated to affect the differences between the perception of crime and actual crime [22]. For example, the elderly, women, and racial and ethnic minorities have been shown as having higher perceptions of crime than actual risk [23, 24]. In the current study, we have a greater percent of elderly female minorities, explaining in part this discrepancy.

When taken into context with other individual factors, this has been referred to as the vulnerability perspective [25]. This idea emphasizes that fear is highest when individuals perceive themselves to be vulnerable [26]. In a 2010 study, investigators examined resident's perception of crime based on the neighborhood in which they live [27]. The results suggested that perceived disorder of neighborhood structure, including social cohesion, was strongly associated with perception of crime even after controlling for race, age, and gender.

In Pittsburgh, Pennsylvania, two large economic developments were recently constructed in two historically disadvantaged minority neighborhoods. In August 2009, gambling was legalized in Pittsburgh, and a casino was opened in the North Side neighborhood. The Consol Energy Center (CEC), an indoor sports and entertainment facility, was opened in the Hill District neighborhood in August 2010. These community-level changes provide a unique opportunity to study the potential effect of two different community economic development efforts to examine whether the economic benefits directly have an effect on perceptions and rates of community violence. Specifically, the current study has three objectives:

1. Describing residents' perceptions of the effect of the arena and casino on neighborhood violence, safety, and economic benefits.
2. Describing residents' perceptions of change in neighborhood violence, safety, and economic benefits after the opening of the arena and casino.
3. Comparing the above residents' perceptions with census and police data over the same time.

Results from this study will fill some of the existing gaps in the field around the relationship between community economic development efforts and community violence while highlighting some of the potential mechanisms through which they may have an effect (e.g., job availability for community residents).

## 2. Methods

### 2.1. Survey Description

**Figure 1:**
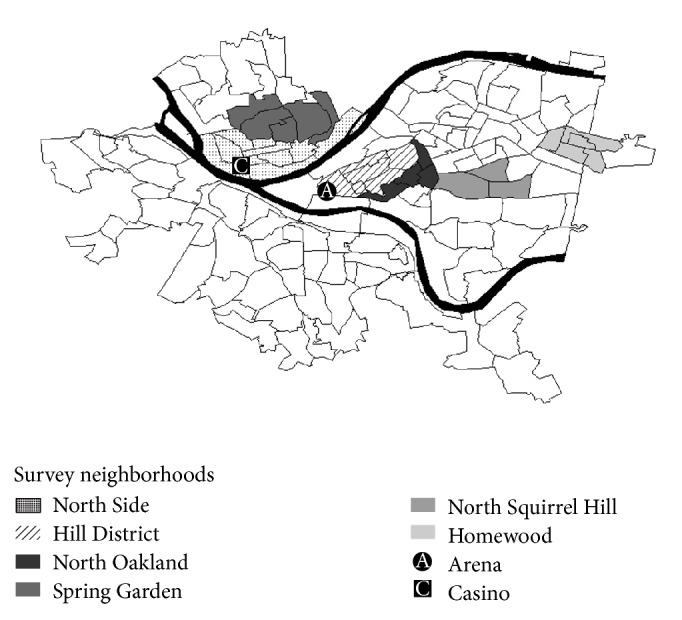
Intervention and comparison areas by 2000 census tracts.

We conducted a telephone survey in 2011 using a listed sample of randomly selected telephone numbers in each of six neighborhoods of Pittsburgh (Figure 1). The neighborhoods included the North Side, where the casino was built in 2009, as well as the Hill District, where the entertainment arena was built in 2010. One adjacent neighborhood for each of these communities was also included to assess whether any potential benefits spilled over into nearby areas. North Oakland was the neighboring community for the Hill District, and an area consisting of Spring Garden, Fineview, Spring Hill-City View, and Perry South (hereafter collectively referred to as Spring Garden) was the neighboring community for the North Side. Two additional neighborhoods were also examined and were intended to serve as comparisons to the economic development communities: Squirrel Hill is a neighborhood of high socioeconomic status (SES) and low violent crime rates, and Homewood is a neighborhood of lower SES and higher levels of violent crime. Interviews were conducted between July and December 2011 and lasted for an average of 25 minutes in length. Survey questions gauged demographic variables, employment history, neighborhood factors, and relationships/interactions with both the casino and the arena. Respondents were categorized by the neighborhood in which they resided based on self-report. In addition, respondents were asked to identify the closest intersection to validate neighborhood assignment.

#### 2.1.1. Perceived Neighborhood Violence

Two measures were used to evaluate perceived neighborhood violence in the communities. One intended to assess the level of violence currently perceived in the neighborhood. Respondents were asked if they felt safe in their neighborhood both during the day and at night. They were also asked if they felt that violence was common in their neighborhood. The values of the three questions were combined into a current perceived violence index. Higher values indicated higher levels of perceived violence.

The second measures gauged the change in perceived violence over the past five years. Respondents were asked whether, compared with 2006, they thought there was more violent crime.

#### 2.1.2. Economic Benefit

Employed respondents were identified as those who were employed for wages/salary, self-employed, student, or a combination of those; unemployed respondents were identified as those who responded “none of the above” to the aforementioned employment selections. Respondents were also asked if their household income had changed since 2006, whether the Rivers Casino affected businesses in their neighborhood, and whether they thought either development affected employment or income in their neighborhood.

### 2.2. Statistical Analysis

The analyses were conducted using SPSS, version 21, software (SPSS Inc., Chicago, Illinois, USA). We conducted descriptive analyses to examine the various measures of perceived violence and safety and economic benefit. We report the number and percent responding to each question. Responses were compared across neighborhoods. Positive and negative responses are shown in the tables. For contrast, we compared the responses with census and police record data. Pearson's chi-square tests determined significant differences in proportions for all neighborhoods. If significant at the *P* < 0.05 level, we then ran pairwise comparisons of column proportions using the Bonferroni correction [34]. We only present comparisons for both the Hill and the North Side compared with each other and the other neighborhoods given that these were our a priori comparisons of interest.

## 3. Results

### 3.1. Sample Demographic (Tables 1 and 2)

Table 1 shows the participants' demographic data. In general, the participants were older than the average age of the surveyed communities, with a mean of 64 ± 15 years of age. The participants were primarily female (69.8%) and white (62.1%), though this varied by neighborhood. Two notable exceptions are the Hill District and Homewood, where around 90% of participants were identified as black (see Table 2 for census figures).

### 3.2. Personal Safety (Table 3)

In Table 3, we show statistically significant differences between the Hill District and the North Side. Squirrel Hill residents were significantly more likely to report feeling safe during the day compared with the Hill and the North Side (99.5% versus 90.6% and 92.4% resp., *P* < 0.05). The percentage of participants in Squirrel Hill who felt safe at night was higher (91.7%, *P* < 0.05), while the percentage in Homewood was lower (48.3%, *P* < 0.05) compared with both the Hill and the North Side. Participants from the Hill District, the North Side, and Spring Garden agreed with the statement “violence is common in my neighborhood” at similar rates (ranging from 32.1 to 42.9%) while far fewer respondents from North Oakland and Squirrel Hill (8.6% and 1.5%, resp., *P* < 0.05) agreed with the statement compared with the Hill and the North Side, and 62.1% of participants from Homewood agreed (*P* < 0.05). Similarly, participants from North Oakland and Squirrel Hill agreed with the statement “compared with 2006, there is more violent crime in my neighborhood now” at the lowest rates (13.7% and 2.4%, resp., *P* < 0.05) compared with the Hill and the North Side and participants from the remaining four neighborhoods agreed at rates ranging from 29.3 to 41.8%.

**Figure 2:**
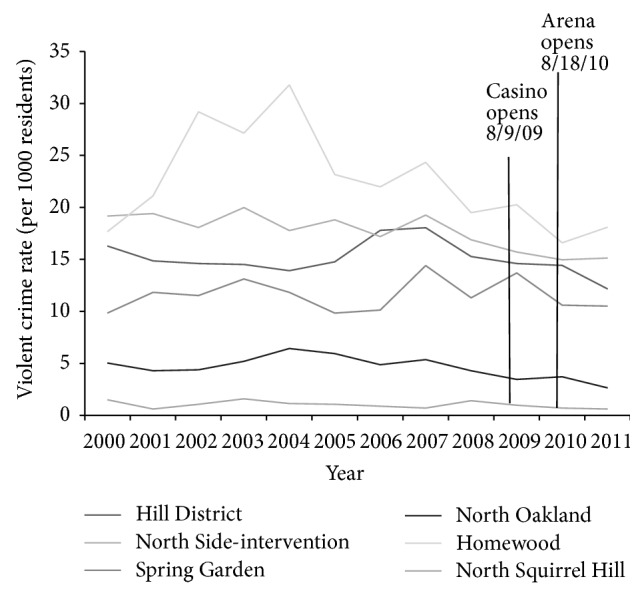
Violent crime rates by survey neighborhood, 2000–2011.

Squirrel Hill had the least amount of recorded violence in 2011 (0.6 violent crimes per 1000 residents), followed by Oakland (2.6), Spring Garden (10.5), Hill District (12.1), North Side (15.1), and Homewood with the most amount of recorded violence (18.1) when looking at actual violent crime rates in these neighborhoods (see Figure 2). All neighborhoods except Spring Garden experienced less violent crime in 2011 than in 2006; Spring Garden saw a slight increase (+0.4).

### 3.3. Household Income Change (Table 3)

No neighborhoods were statistically different from either the Hill or the North Side.

### 3.4. Perceived Effect of Rivers Casino (Table 4)

Participants in the North Side stated more often that the Rivers Casino has had a positive impact on their life (14.1%) compared with 7.2% of participants from the North Side who responded that the casino has had a negative impact. Participants in Squirrel Hill were more likely to not report positive effects compared with the North side (*P* < 0.05). Participants from both North Oakland and Squirrel Hill were statistically much less likely to report an effect on economic issues compared with the North Side (*P* < 0.05, Table 3). When asked, “how has the Rivers Casino affected crime in your neighborhood?,” the only statistical difference was for Squirrel Hill, with no one reporting that the casino had a positive effect on crime in his or her neighborhood (*P* < 0.05).

In summary, the North Side appears to have benefitted more from the casino than any other neighborhoods, but this benefit was only reported by a minority of North Side participants. This is reflected in responses to the question “overall, how has the Rivers Casino affected your neighborhood?” The highest rate of responses indicating a positive effect was observed in the North Side, at 24.5%, while the majority of participants in each neighborhood responded “neither positively nor negatively” (58.6% of participants in the North Side). The less disadvantaged neighborhoods, North Oakland and Squirrel Hill, were the only neighborhoods with significant differences.

### 3.5. Perceived Effect of Consol Energy Center (Table 5)

When asked, “how has the Consol Energy Center impacted your life?,” the majority of participants in each neighborhood answered “neither positively nor negatively” (69.6% of participants from the Hill District, where the arena is located, and around 80% of participants in all other neighborhoods). When asked, “how has the Consol Energy Center affected income in your neighborhood?,” the most common answer across all neighborhoods was “neither positively nor negatively” (44.4% of participants in the Hill District, location of the CEC). Responses to the other economic questions followed a similar pattern. 31.9% of participants from the Hill District reported that the Consol Energy Center had a positive effect on employment in their neighborhood, and 24.6% of participants from the Hill District reported that the Consol Energy Center had a positive effect on local businesses in their neighborhood. Participants from every other neighborhood reported statistically significantly lower proportions of positive effects on the economic questions compared to the Hill (*P* < 0.05).

In response to the question “how has the Consol Energy Center affected crime in your neighborhood?,” the majority of participants in each neighborhood responded “neither positively nor negatively” (68.6% of participants from the Hill District), followed by the response “do not know/unsure” (18.8% of participants from the Hill District). Responses to the question “how has the Consol Energy Center affected violence in your neighborhood?” followed a similar pattern, with 68.6% of participants from the Hill District responding “neither positively nor negatively” and 17.4% of participants from the Hill District responding “do not know/unsure.”

In summary, participants from the Hill District reported benefitting the most from the Consol Energy Center. When asked, “overall, how has the Consol Energy Center affected your neighborhood?,” 32.9% of participants from the Hill District reported that the arena had a positive effect while 46.4% of respondents reported that the arena had neither a positive nor a negative effect, and 9.7% reported a negative effect. Participants in all other neighborhoods reported statistically significantly lower levels of positive effects in comparison (*P* < 0.05).

## 4. Discussion

Many factors influence the prevalence of youth violence. Community economic development is one strategy that may lead to more resources and opportunities within neighborhoods. This, in turn, may result in reductions in community-level rates of violence. This study addressed the relationship between two large economic development efforts within the city of Pittsburgh—a casino and a sports arena—on perceptions of economic opportunities and community safety. Overall, we found that residents in neighborhoods with the large-scale economic developments reported more development in specific economic benefits than did residents from other neighborhoods. A large proportion of Hill District respondents thought violent crime had increased since 2006 even though it actually decreased more in the Hill District than in any other survey neighborhoods. In addition, Hill District respondents felt less safe than North Side respondents did, even though the North Side experiences more crime than the Hill District. However, the crime rates are calculated per residents. If more crime in the North Side is being committed by nonresidents in the entertainment districts than crime in the Hill District being committed by nonresidents coming into the Hill, the North Side residents may actually be experiencing less violence near their homes than Hill District residents.

When participants' perceptions are compared with the actual violent crime rates for their neighborhoods, they often matched. Participants from Squirrel Hill were most likely to say they felt safe during the day or night and were least likely to believe that violence is common in their neighborhood while participants from Homewood were least likely to say they felt safe during the day or night and were most likely to believe that violence is common in their neighborhood. Accordingly, the 2011 violent crime rates indicate that Squirrel Hill experienced the least amount of violence (0.6 violent crimes per 1000 residents) of the six neighborhoods survey while Homewood experienced the most (18.1 violent crimes per 1000 residents). It should be noted, however, that although the majority of participants from Homewood agreed that violence is common in their neighborhood and indeed it is thirty times more common in Homewood than Squirrel Hill, the majority of participants did not feel unsafe, even at night. Participants were also mostly correct in their perceptions about the change in violence since 2006. The majority from every neighborhood did not believe that there was more violence now than in 2006, and indeed violent crime rates have decreased in every neighborhood except Spring Garden, which saw a slight increase. Interestingly, however, the rate of violent crime in Spring Garden in 2011 was at its lowest since 2006 due to a spike in 2007, so it may have been difficult for participants in that neighborhood to differentiate between the slight increase in violence compared with 2006 and the decrease in violence seen almost every year since 2007. Even though Homewood and the Hill District had the greatest declines in violence from 2006 to 2011 of all survey neighborhoods, this was not evident in the participant responses in these neighborhoods.

When the perception of crime is taken into context with other individual factors, it has been referred to as the vulnerability perspective [25]. This idea emphasizes that fear is highest when individuals perceive themselves to be vulnerable [26]. In a 2010 study, investigators examined resident's perception of crime based on the neighborhood in which they live [27]. The results suggested that perceived disorder of neighborhood structure, including social cohesion, was strongly associated with perception of crime even after controlling for race, age, and gender.

Two major criminological theories on crime, the broken windows hypothesis [28] and the collective efficacy perspective [29, 30], are related to both actual crime and the perception of crime. Neighborhood structure, such as social cohesion, can influence perception of crime. Social cohesion measures mutual trust among residents and is thought to reduce community problems including fear of crime. Previous studies have shown that concentrated disadvantage within communities, as measured by poverty, unemployment, and family disruption, is associated with fear of crime [26, 29, 31]. As in our study, low levels of social cohesion may lead to more crime [28, 32], which may in turn reduce cohesion among residents [33]. There are likely differences in our results between perception and reality of crime because of individual characteristics, including age, gender, and race, as well as neighborhood context, which has been shown to affect individuals' perception of crime.

Surprisingly, there has been a dearth of research examining whether casinos positively benefit community residents [10]. The few studies that have assessed economic growth looked at large geographic areas, such as county or state, rather than areas directly surrounding the developments. An evaluation of the economic impact of casinos by Eadington used historical perspective to demonstrate the potential economic benefits [14]. The author cited Monaco, Nevada, and Atlantic City as places that, before the commercialization of legalized gambling, were experiencing economic deterioration. However, after the licensing of casinos in these areas, they economically prospered and became destinations for tourism. Other groups studied the causal impact of casino gambling profits and per capita income and found that there was no evidence for causation of casino profits impacting per capita income in two studies [10, 35]. Both evaluations were conducted at a state level where data were aggregated for all 11 states that allowed casino gambling on any level. The impact on smaller geographic areas is not well known [35]. Our results begin to fill this gap in the literature by examining residents' specific perceptions of the impact of area developments and providing a glimpse into how the developments affect individuals as well as communities.

Grinols argues that economic development occurs when welfare or utility increases within a neighborhood [9]. However, others theorize that economic growth often “leaks” outside of the community as profits and jobs are exported out of the community [9, 10]. Our results are more in line with Grinols' research. We found that the highest rate of respondents stating that the Rivers Casino has had a positive impact on their life was in the neighborhood where the casino is located; a similar pattern was observed for the neighborhood in which the arena is located. Additionally, the results of the economic benefit questions underlined these findings. Residents of the neighborhoods in which the casino and arena are located reported the greatest positive effects on their income.

There are a large number of studies that have assessed the effect of large-scale developments on violence. However, these are mostly related to casinos, and the results are varied. In a study by Grinols and Mustard, casino and noncasino counties were evaluated after the construction and opening of casinos [16]. Both violent and property crime levels were evaluated for two years before the opening and five years following the opening. The results of the study showed that there was an increase in crime after the opening of a casino. Further, Park and Stokowski compared four types of counties based on predominant type of recreation/tourism attraction offered, including casinos, and found that total arrest rate was highest in casino counties but this was not statistically significant [36]. Stokowski found that gaming counties in Colorado had higher rates of property crimes but not violent crimes. Research has also indicated that economic crimes and public order crimes increased in Biloxi, MS, after introduction of casino gaming [15, 37], and disorderly conducted arrests increased after introduction of casino gaming in Davenport, Iowa [38]. Our results, while limited to residents' perceptions of violence, further support the finding that violence increases after the opening of large-scale developments, in this case a casino. We found that residents in the neighborhoods with the developments reported greater changes in violence. The differences in results across studies are likely caused by differences in measures of violence and size of the geographic area studied. More research is needed in this area with particular emphasis on using consistent definitions, measures, and methodology.

Interestingly, in the Grinols and Mustard study, the increase in violent crime occurred most noticeably after a three-year lag [16]. The investigators postulated that this was likely a result of two factors. First, in the initial years following the opening of a casino, more community resources, such as funding for labor of police, are at their highest and this in turn reduces crime. Additionally, the investigators cite data concerning the effects of addictive gamblers [16]. Our results do not contain data after 2 years for the casino and 1 year for the arena, but these findings provide important implications to consider in future research.

*Study Limitations*. There is a large amount of heterogeneity between communities which demonstrates the need to assess how casinos may affect different types of neighborhoods differently. For example, Kang et al. studied residents' perceptions of the impact of limited-stakes community-based casino gambling in Colorado in 2006 [39]. Perceived positive economic impacts from the casino influenced residents' support of the casino, while perceived negative social impacts did not. It is important to assess how the effects vary by community and individual. Our sample consisted largely of older women, and our findings may not be representative of all age groups, particularly young men and women who may be more likely to visit, attend events at, and work at the casino and arena, likely due in part to only including landlines. Additionally, we did not assess actual changes in neighborhood opportunities. Further research should examine objective data in combination with subjective data regarding residents' perceptions. Finally, our analyses are solely descriptive. More nuanced work should be done to assess independent effects on subgroups.

This initial work is important in identifying how neighborhood residents perceive the large-scale economic developments in these neighborhoods. The potential benefits to a community are directly related to public health outcomes as well as public policy. More in-depth studies should be conducted to assess long-term effects as well as the social pathways through which these effects occur.

## Figures and Tables

**Table 1:** Demographic characteristics of study population from survey responses.

	Hill-intervention (*n* = 226)	North Side (*n* = 268)	Spring Garden (*n* = 173)	Homewood (*n* = 179)	North Oakland (*n* = 218)	Squirrel Hill (*n* = 209)
Gender						
Male	46 (20.4%)	94 (35.1%)	50 (28.9%)	50 (27.9%)	70 (32.1%)	69 (33.0%)
Female	180 (79.6%)	174 (64.9%)	123 (71.1%)	129 (72.1%)	148 (67.9%)	140 (67.0%)
Age (SD)	60.3 (16.9)	60.8 (15.5)	60.2 (16.0)	62.2 (17.0)	69.7 (16.8)	62.0 (15.7)
Race						
White	16 (7.2%)	159 (60.0%)	128 (75.3%)	10 (5.8%)	173 (80.8%)	195 (93.3%)
Black	204 (91.5%)	95 (35.9%)	40 (23.5%)	157 (90.8%)	35 (16.4%)	4 (1.9%)
Others	3 (1.4%)	11 (4.2%)	2 (1.2%)	6 (3.5%)	6 (2.8%)	10 (4.8%)
Education						
<HS degree	14 (6.3%)	21 (7.9%)	9 (5.2%)	10 (5.6%)	10 (4.6%)	1 (0.5%)
HS degree^*∗*^	81 (36.3%)	86 (32.2%)	82 (47.4%)	74 (41.6%)	24 (11.0%)	7 (3.4%)
>HS degree	128 (57.4%)	160 (59.9%)	82 (47.4%)	94 (52.8%)	184 (84.4%)	201 (96.2%)
Employment						
Employed	60 (26.9%)	99 (36.9%)	67 (38.7%)	52 (29.2%)	49 (22.7%)	86 (41.8%)
Not employed	163 (73.1%)	169 (63.1%)	106 (61.3%)	126 (70.8%)	167 (77.3%)	120 (58.3%)
Marital status						
Married	122 (54.5%)	117 (43.8%)	51 (29.8%)	84 (47.5%)	70 (32.1%)	31 (14.9%)
Unmarried	102 (45.5%)	150 (56.2%)	120 (70.2%)	93 (52.5%)	148 (67.9%)	177 (85.1%)

All data presented as number with percentage of total in parentheses. SD = standard deviation for continuous measure of age. ^*∗*^HS degree or equivalent.

**Table 2:** Demographic characteristics of study neighborhood from the 2010 Census or 2006–2011 ACS.

	Hill-intervention (*n* = 226)	North Side (*n* = 268)	Spring Garden (*n* = 173)	Homewood (*n* = 179)	North Oakland (*n* = 218)	Squirrel Hill (*n* = 209)
Total population	9754	12252	8038	6279	12016	12378
% black	88.4	44.5	50.9	96	17	3.5
% male	42.9	49.7	44.8	40.7	45.8	52.6
% unemployed male	7.9	7.2	8.8	7.3	2.7	2.3
% families in poverty	38.7	23.5	31.8	35	15.7	1.3
% households female-headed	26.7	17.3	26.2	29.5	8.2	5.1
% high school education or GED	83.1	86.7	84.8	86.2	94	97.7
Average median household income^*∗*^	17465	31573	25900	23862	21325	86169

^*∗*^Calculated as the average of the median incomes by census tract in the neighborhood.

**Table 3:** Personal safety and household income change since 2006 by neighborhood.

	Hill, arena location	North Side, casino location	North Oakland	Spring Garden	Homewood	Squirrel Hill
	I feel safe in my neighborhood during the day					
Agree	192 (90.6%)	232 (92.4%)	203 (96.7%)	153 (91.1%)	146 (83.9%)	203 (99.5%)^*∗*,#^
Disagree	14 (6.6%)	12 (4.8%)	2 (1.0%)^*∗*^	12 (7.1%)	22 (12.6%)^#^	0^*∗*,#^
*n*	212	251	210	174	174	204

	I feel safe in my neighborhood at night					
Agree	136 (64.2%)	161 (64.1%)	137 (65.2%)	105 (62.5%)	84 (48.3%)^*∗*,#^	187 (91.7%)^*∗*,#^
Disagree	58 (27.4%)	67 (26.7%)	46 (21.9%)	52 (31.0%)	75 (43.1%)^*∗*,#^	7 (3.4%)^*∗*,#^
*n*	212	251	210	168	174	204

	Violence is common in my neighborhood					
Agree	91 (42.9%)	85 (34.0%)	18 (8.6%)^*∗*,#^	54 (32.1%)	108 (62.1%)^*∗*,#^	3 (1.5%)^*∗*,#^
Disagree	106 (50.0%)	139 (55.6%)	177 (84.3%)^*∗*,#^	103 (61.3%)	48 (27.6%)^*∗*,#^	196 (96.1%)^*∗*,#^
*n*	212	250	210	168	174	204

	Compared to 2006, there is more violent crime in my neighborhood now					
Agree	66 (41.8%)	56 (29.3%)	21 (13.7%)^*∗*,#^	47 (35.1%)	59 (40.7%)	4 (2.4%)^*∗*,#^
Disagree	76 (48.1%)	111 (58.1%)	102 (66.7%)^*∗*^	73 (54.5%)	60 (41.4%)^#^	135 (80.8%)^*∗*,#^
*n*	158	191	153	134	145	167

	Household income change from 2006					
Increased	53 (24.3%)	85 (32.7%)	49 (23.1%)	57 (33.9%)	56 (32.0%)	67 (34.7%)
Decreased	60 (27.5%)	71 (27.3%)	66 (31.1%)	39 (23.2%)	38 (21.7%)	51 (26.4%)
*n*	218	260	212	168	175	193

^*∗*^Statistically different from the Hill District.

^#^Statistically different from the North Side.

**Table 4:** Perceived effect of Rivers Casino on neighborhood.

	Hill, arena location	North Side, casino location	North Oakland	Spring Garden	Homewood	Squirrel Hill
	How has the Rivers Casino impacted your life?					
Positively	22 (10.6%)	35 (14.1%)	18 (8.6%)	16 (9.5%)	16 (9.3%)	4 (2.0%)^#^
Negatively	10 (4.8%)	18 (7.2%)	7 (3.3%)	13 (7.7%)	10 (5.8%)	11 (5.4%)
*n*	208	249	209	168	172	204

	How has the Rivers Casino affected employment in your neighborhood?					
Positively	39 (18.8%)	65 (26.1%)	7 (3.3%)^#^	26 (15.5%)	16 (9.3%)^#^	4 (2.0%)^#^
Negatively	14 (6.7%)	11 (4.4%)	3 (1.4%)	4 (2.4%)	3 (1.7%)	2 (1.0%)
*n*	208	249	209	168	172	204

	How has the Rivers Casino affected income in your neighborhood?					
Positively	24 (11.5%)	28 (11.2%)	8 (3.8%)^#^	23 (13.7%)	10 (5.8%)	1 (0.5%)^#^
Negatively	22 (10.6%)	16 (6.4%)	4 (1.9%)	4 (2.4%)	2 (1.2%)	2 (1.0%)^#^
*n*	208	249	209	168	172	204

	How has the Rivers Casino affected businesses in your neighborhood?					
Positively	22 (10.6%)^#^	61 (24.5%)	9 (4.3%)^#^	21 (12.5%)^#^	6 (3.5%)^#^	1 (0.5%)^#^
Negatively	12 (5.8%)	9 (3.6%)	4 (1.9%)	5 (3.0%)	4 (2.3%)	2 (1.0%)
*n*	208	249	209	168	172	204

	How has the Rivers Casino affected crime in your neighborhood?					
Decreased	13 (6.3%)	9 (3.6%)	5 (2.4%)	5 (3.0%)	5 (2.9%)	0
Increased	12 (5.8%)	13 (5.2%)	5 (2.4%)	10 (6.0%)	3 (1.7%)	3 (1.5%)
*n*	208	249	209	168	172	204

	How has the Rivers Casino affected violence in your neighborhood?					
Decreased	8 (3.8%)	9 (3.6%)	4 (1.9%)	3 (1.8%)	5 (2.9%)	0
Increased	10 (4.8%)	10 (4.0%)	4 (1.9%)	7 (4.2%)	2 (1.2%)	3 (1.5%)
*n*	208	249	209	168	172	204

	Overall, how has the Rivers Casino affected your neighborhood?					
Positively	32 (15.4%)	61 (24.5%)	10 (4.8%)^#^	24 (14.3%)	17 (9.9%)^#^	2 (1.0%)^#^
Negatively	15 (7.2%)	15 (6.0%)	6 (2.9%)	6 (3.6%)	4 (2.3%)	4 (2.0%)
*n*	208	249	209	168	171	204

^#^Statistically different from the North Side.

**Table 5:** Perceived effect of Consol Energy Center on neighborhood.

	Hill, arena location	North Side, casino location	North Oakland	Spring Garden	Homewood	Squirrel Hill
	How has the Consol Energy Center impacted your life?					
Positively	35 (16.9%)	28 (11.2%)	19 (9.1%)	18 (10.7%)	18 (10.5%)	29 (14.2%)
Negatively	23 (11.1%)	7 (2.8%)^*∗*^	12 (5.7%)	5 (3.0%)^*∗*^	4 (2.3%)^*∗*^	4 (2.0%)^*∗*^
*n*	207	249	209	168	172	204

	How has the Consol Energy Center affected employment in your neighborhood?					
Positively	66 (31.9%)	29 (11.6%)^*∗*^	15 (7.2%)^*∗*^	17 (10.1%)^*∗*^	18 (10.5%)^*∗*^	2 (1.0%)^*∗*^
Negatively	12 (5.8%)	6 (2.4%)	2 (1.0%)	2 (1.2%)	1 (0.6%)	0^*∗*^
*n*	207	249	209	168	172	204

	How has the Consol Energy Center affected income in your neighborhood?					
Positively	56 (27.1%)	27 (10.8%)^*∗*^	11 (5.3%)^*∗*^	8 (4.8%)^*∗*^	11 (6.4%)^*∗*^	3 (1.5%)^*∗*^
Negatively	11 (5.3%)	6 (2.4%)	2 (1.0%)	3 (1.8%)	1 (0.6%)	0^*∗*^
*n*	207	249	209	168	172	204

	How has the Consol Energy Center affected local businesses in your neighborhood?					
Positively	51 (24.6%)	28 (11.2%)^*∗*^	11 (5.3%)^*∗*^	7 (4.2%)^*∗*^	5 (2.9%)^*∗*^	8 (3.9%)^*∗*^
Negatively	12 (5.8%)	3 (1.2%)	3 (1.4%)	3 (1.8%)	3 (1.7%)	1 (0.5%)^*∗*^
*n*	207	249	209	168	172	204

	How has the Consol Energy Center affected crime in your neighborhood?					
Decreased	17 (8.2%)	4 (1.6%)^*∗*^	4 (1.9%)	2 (1.2%)^*∗*^	3 (1.7%)	2 (1.0%)^*∗*^
Increased	9 (4.3%)	5 (2.0%)	6 (2.9%)	3 (1.8%)	3 (1.7%)	0^*∗*^
*n*	207	249	209	168	172	204

	How has the Consol Energy Center affected violence in your neighborhood?					
Decreased	19 (9.2%)	2 (0.8%)^*∗*^	4 (1.9%)^*∗*^	2 (1.2%)^*∗*^	3 (1.7%)^*∗*^	1 (0.5%)^*∗*^
Increased	10 (4.8%)	7 (2.8%)	7 (3.3%)	4 (2.4%)	3 (1.7%)	1 (0.5%)
*n*	207	249	209	168	172	204

	Overall, how has the Consol Energy Center affected your neighborhood?					
Positively	68 (32.9%)	34 (13.7%)^*∗*^	20 (9.6%)^*∗*^	13 (7.7%)^*∗*^	12 (7.0%)^*∗*^	11 (5.4%)^*∗*^
Negatively	20 (9.7%)	5 (2.0%)^*∗*^	8 (3.8%)	3 (1.8%)^*∗*^	5 (2.9%)	1 (0.5%)^*∗*^
*n*	207	249	209	168	172	204

^*∗*^Statistically different from the Hill District.
